# Perceptions, Attitudes, and Behaviors Related to Hepatitis Among the General Population Attending a COVID-19 Vaccination Center in a Tertiary Care Hospital in India

**DOI:** 10.7759/cureus.65327

**Published:** 2024-07-25

**Authors:** Manya Prasad, Aayushi Rastogi, Mini George, Iswarya S, Sarita Ahwal, Sumridhi Gautam, Priyanka Aggarwal

**Affiliations:** 1 Clinical Research and Epidemiology, Institute of Liver and Biliary Sciences, New Delhi, IND; 2 College of Nursing, Institute of Liver and Biliary Sciences, New Delhi, IND

**Keywords:** india, : vaccination, public health, knowledge attitude and practice, knowledge, hepatitis-c, hepatitis-b

## Abstract

Background and objective

Hepatitis B and C are major public health issues in India, associated with significant morbidity and mortality. Understanding the general population's perceptions, values, attitudes, and behaviors regarding these infections is essential for effective health interventions. In light of this, the present study aimed to assess the knowledge, attitude, and practice (KAP) related to hepatitis B and C among individuals attending a coronavirus disease 2019 (COVID-19) vaccination center at a tertiary care hospital in India.

Methods

A cross-sectional survey involving 759 participants was conducted using a structured questionnaire. Data on demographics and hepatitis-related KAP were collected via face-to-face interviews. Descriptive statistics, chi-square tests, multivariate logistic regression, and Pearson’s correlation coefficients were used for data analysis.

Results

The mean age of the participants was 33.4 years, with 445 (58.6%) of them being males. Most had at least a high school education, and 467 (61.53%) were employed. The mean knowledge score was 18.94 out of a possible total score of 45. While 529 (69.7%) knew that hepatitis affects the liver, only 317 (41.77%) were aware of the various types of viral hepatitis. The mean attitude score was 15.28 out of 21, with 78.73% willing to seek treatment if diagnosed. However, only 133 (17.55%) believed they were at risk of contracting hepatitis B. The mean practice score was 7.95 out of 15. About 256 (33.73%) had completed the hepatitis B vaccination course and 186 (24.5%) had been screened for hepatitis B or C.

Conclusions

The study indicates moderate knowledge but significant gaps in understanding about hepatitis transmission and prevention among the general public. Attitudes towards vaccination and treatment are generally positive, but practices related to prevention are inconsistent. Enhanced public health education and targeted interventions are needed to improve hepatitis-related KAP among the general population.

## Introduction

Hepatitis is an inflammatory disease of the liver caused by a range of viral and non-infectious factors leading to several health complications, some of which are potentially fatal [1]. Worldwide, hepatitis B virus (HBV) and hepatitis C virus (HCV) are some of the leading causes of liver-related mortality, cirrhosis, liver cancer, and liver transplantation [2]. According to estimates by the World Health Organization (WHO), in 2019, there were 296 million cases of hepatitis B, 58 million cases of hepatitis C, 1.5 million new cases of chronic hepatitis B, and 1.5 million new cases of chronic hepatitis C infections worldwide [3]. The disease is the second most common infectious cause of death worldwide, accounting for 1.3 million deaths annually, second only to tuberculosis [4].

According to the most recent estimates, 40 million individuals in India have a chronic hepatitis B infection, and 6-12 million have a chronic hepatitis C infection [5]. The available interventions to combat these conditions include widespread HBV immunization campaigns, HBV and HCV screening and care linkage, preventing mother-to-child transmission, encouraging safe injection techniques, strict infection control initiatives, and antiviral therapies for individuals with HBV and HCV infections [6]. The increased prevalence of HBV and HCV in developing nations may be caused by several significant factors, such as tattooing, unsafe sexual practices (polygamy), reusing unsterilized syringes, accidental needlestick injuries in hospital settings, intravenous drug addiction, unsafe blood transfusion procedures, vertical transmission to the unborn child, etc. [7].

The Global Health Sector Strategy on Viral Hepatitis 2016-2021 (GHSSH) aims to eradicate viral hepatitis by 2030, aiming for a 90% reduction in incidence and 65% reduction in mortality [8]. Also, Target 3.3 of SDG 3 addresses fighting viral hepatitis and intends to eliminate the disease by 2030 [9]. The National Viral Hepatitis Control Program in India aims to eliminate hepatitis C by 2030 and reduce the infected population, morbidity, and mortality associated with hepatitis B and C, including cirrhosis and hepatocellular carcinoma [10]. Given the diversity of cultures, religions, and belief systems in developing nations such as India, it is critical to ascertain the degree of awareness that different societal segments have regarding HBV and HCV. Limited awareness regarding the prevention of HBV has been reported by previous studies among the general population in India. Moreover, vaccination rates, especially for the crucial birth doses, are reported to be low. To address these issues, it is imperative to implement robust public health initiatives [11].

The healthcare system's ability to control hepatitis can be strengthened by increased public awareness of the disease through organized planning, the implementation of various health awareness initiatives, and legislation. In India, efforts are being made to raise awareness about hepatitis B and C and their prevention through various awareness programs and media. Understanding the knowledge, attitude, and practice (KAP) regarding these infections among the general population is crucial for designing effective health interventions. This study aims to assess the KAP concerning hepatitis among individuals attending a coronavirus disease 2019 (COVID-19) vaccination center in a tertiary care hospital in India.

## Materials and methods

Study design and participants

This cross-sectional survey was conducted during the COVID-19 pandemic among the general public who attended a COVID-19 vaccination center at a tertiary care hospital in India from September 2021 to January 2022. The participants were selected by the consecutive sampling of all individuals attending the center. The inclusion criteria were as follows: all adults aged 18 years who had come for any dose of the COVID-19 vaccine at the time of data collection and gave consent to participate.

Data collection

A pre-devised, close-ended structured questionnaire which was pre-tested was developed based on previous literature. The questionnaire had four sections, including subjects’ sociodemographic and educational information as well as their KAP related to hepatitis B and C.

Survey Questionnaire

The participants completed the questionnaire at the vaccination center through face-to-face interviews, administered by healthcare professionals. The queries of the public were also addressed by the administrator after they completed the interview. The knowledge section contained 30 questions covering four domains of general overview of disease, causes and transmission, prevention and treatment strategies for hepatitis B and C; 30 questions were categorized into four domains of knowledge. Domain 1 (D1) had four questions related to a general overview of the disease. Domain 2 (D2) included two questions about the modes of transmission of hepatitis B and C. Domain 3 (D3) and Domain 4 (D4) had three questions each about the vaccination, prevention, and treatment of the disease.

The awareness section had seven questions with binomial outcomes of "yes" and "no" relating to self and family’s hepatitis infection and vaccination status. The attitude section included 5-point Likert-like questions with responses including "strongly disagree", "disagree", "neutral", "agree", and "strongly agree" choices. The practice section contained five questions with responses of "never", "rarely", "sometimes", and "often" as options for preventive strategies and as "yes", "no", and "not sure" for screening and vaccination strategies.

A score of 1 was awarded for a correct response in the knowledge and practice sections, and the attitude section and the sum of the individual scores were considered. The total score ranged from 0 to 45 for knowledge, 0 to 21 for attitude, and 0 to 15 for practice. We established cut-off points for knowledge, awareness, and practices by using a combination of expert consultations and reviewing existing literature to ensure they were contextually relevant and accurately reflected the population's understanding and behaviors. To assess the internal consistency and reliability of the KAP survey tool, Cronbach's alpha was calculated, yielding a value of 0.75, indicating acceptable reliability for the survey instrument. A total score of ≥23 was considered good knowledge, 12-22 as fair knowledge, and 0-11 as poor knowledge A score of ≥11 was defined as a positive attitude, and a score of ≤7 as a negative attitude. A score of ≥8 was taken as safe practice and less than 8 as unsafe practice.

Ethical consideration

The study was approved by the Institutional Ethics Committee of the Institute of Liver and Biliary Sciences (approval no: F.37/(1)/9/ILBS /DOA /2020/20217/351)

Statistical analysis

Data collected were analyzed using IBM SPSS Statistics for Windows, Version 27 (IBM Corp., Armonk, NY). A p-value <0.05 was considered statistically significant. A descriptive analysis was initially conducted, which was followed by a normality test. Continuous variables were represented in the form of mean and standard deviation (SD), and categorical variables were depicted in frequency and proportions. A chi-square test and logistic regression analysis were conducted to determine the association of independent variables with the outcome variable of interest (knowledge, attitude, and practice related to hepatitis B and C). Variables yielding a p-value of <0.20 in the bivariable analysis were included in the multivariable analysis. Pearson’s correlation coefficients were used to determine the correlation between knowledge, awareness, attitude, and practice.

## Results

Distribution of the respondents by sociodemographics

The mean age of the participants was 33.4 ± 11.5 years, and the majority of the respondents were males (58.6%). Regarding their education status, 296 (39%) were graduates and 150 (20%) had completed postgraduate education. The majority (61.53%) of the participants were employed and working in the private sector (35.84%). Most of the study participants belonged to the first class (54.28%) in terms of their socioeconomic status as per BG Prasad socioeconomic classification 2021; 467 (61%) participants were staying in nuclear families (Table 1).

**Table 1:** Demographic characteristics of the study participants (N=759) SD: standard deviation

Demographic characteristics	Values
Age in years, mean ± SD	33.4 ± 11.5
Gender, n (%)	
Male	445 (58.6)
Education, n (%)	
Illiterate	12 (1.58)
Upto class 12th	98 (12.9)
Intermediate/class 12th	102 (13.44)
Diploma	79 (10.41)
Graduate and above	468 (61.6)
Employment status, n (%)	
Dependent	74 (9.75)
Employed	467 (61.53)
Unemployed	218 (28.7)
Sector of Job	
Dependent	87 (11.46)
Unemployed	101 (13.31)
Self-employed	45 (5.93)
Government	228 (30.04)
Private	272 (35.84)
Public	26 (3.43)
Social-economic status (as per BG Prasad classification 2021), ₹, n (%)	
Class I (>7770)	412 (54.28)
Class II (3808–7770)	129 (17)
Class III (2253–3808)	108 (14.23)
Class IV (1166–2253)	67 (8.83)
Class V (<1166)	43 (5.67)
Marital status, n (%)	
Unmarried	344 (45.32)
Married	405 (53.36)
Divorced/separated/widowed	10 (1.32)
Type of family, n (%)	
Living alone	45 (5.93)
Nuclear	467 (61.53)
Joint	236 (31.09)
Extended	11 (1.45)

Overall scores of the respondents

Table 2 shows the summary of KAP scores among the respondents. The mean scores for different factors were as follows - knowledge: 18.94 ± 5.65; attitude: 15.28 ± 4.13; and practice: 7.95 ± 2.94. The respondents had a median score of 3 with an interquartile range (IQR) of 1-4 in the general domain of knowledge, a score of 10 (7-12) in the domain of mode of transmission, a score of 3 (2-4) in prevention, and 4 (3-5) in last domain of complication and treatment.

**Table 2:** Overall scores for knowledge, attitude, and practice related to viral hepatitis among the study participants (N=759) IQR: interquartile range; SD: standard deviation

Knowledge, attitude, and practice	Score
Domain 1 (general overview), median (IQR)	3 (1-4)
Domain 2 (mode of transmission), median (IQR)	10 (7-12)
Domain 3 (prevention), median (IQR)	3 (2-4)
Domain 4 (complication and treatment), median (IQR)	4 (3-5)
Overall scores, mean (SD)	
Knowledge	18.94 (5.65)
Attitude	15.28 (4.13)
Practice	7.95 (2.94)

Level of knowledge, attitude, and practice

As per the survey of the knowledge component (Table 3), 69.7% knew that hepatitis B and C primarily affect the liver, but only 41.77% were aware of the different types of viral hepatitis. Knowledge about modes of transmission varied, with high awareness regarding transmission through blood products (83%) and awareness of vertical transmission, i.e., from infected mother to fetus (70.22%). Moreover, the majority of the participants (81.82%) were aware that hepatitis B and C could spread via tattooing by using contaminated needles or ink. Similarly, 75.49% of individuals knew that hepatitis B could result in cirrhosis or hepatocellular cancer if not treated in time. Among the respondents, 79.18% were aware that hepatitis B could be prevented by getting vaccinated. However, only 35.7% were aware of the hepatitis B vaccination schedule for adults.

**Table 3:** Correct responses to knowledge-related questions among the study participants (N=759)

Sl. no.	Domain	Knowledge-related questions	Correct responses, n (%)
K1	D1	Which organ does hepatitis B and hepatitis C mainly affect?	529 (69.7)
K2	D1	How many common types of viral hepatitis are there?	317 (41.77)
K3	D1	What is the cause of hepatitis B and C?	451 (59.42)
K4-12	D2	How do hepatitis B and C spread?	
		Contaminated water and food	395 (52.04)
		Infected blood and blood products	630 (83)
		Handshaking and hugging	536 (70.62)
		Tattooing and sharing razors	567 (74.7)
		Sexual contact with an infected person	593 (78.13)
		From infected mother to fetus	533 (70.22)
		Reuse of syringes and needles	600 (79.05)
		Surgical/dental procedure	526 (69.3)
		Mosquito/insect bite	568 (74.84)
K13-16	D2	How do Hepatitis B and C infections spread by tattooing?	
		Use of colorful inks	576 (75.89)
		Use of contaminated needles and/or ink	621 (81.82)
		Consuming contaminated food and water at the tattoo parlor	405 (53.36)
		Inhaling the dust accumulated in the tattoo parlor	535 (70.49)
K17	D1	What are the most common signs and symptoms of acute hepatitis B infection?	463 (61)
K18-22	D4	Which of the following infections can result in cirrhosis or hepatocellular cancer if not treated in time?	
		Hepatitis A	491 (64.69)
		Hepatitis B	573 (75.49)
		Hepatitis C	549 (72.33)
		Hepatitis D	318 (41.9)
		Hepatitis E	485 (63.9)
K23	D4	Which of the following infections is curable?	240 (31.62)
K24	D4	Treatment for the following needs to be taken lifelong as the disease is not curable	396 (52.17)
K25-28	D3	Which of the following infections can be prevented by getting vaccinated?	
		Hepatitis A	372 (49.01)
		Hepatitis B	601 (79.18)
		Hepatitis C	389 (51.25)
		Hepatitis E	498 (65.61)
K29	D3	How many doses does a complete vaccination for hepatitis B require?	344 (45.32)
K30	D3	Hepatitis B vaccine schedule among adults is	271 (35.7)

Attitude toward hepatitis B and C status

Respondents were asked seven questions about their attitude toward the disease (Table 4). A significant proportion (40.5%) expressed willingness to seek further investigation and treatment if diagnosed with hepatitis B. However, only 17.55% strongly believed they could acquire hepatitis B. About 35.36% of the individuals agreed that hepatitis B vaccination is safe. Around 44% of the respondents were neutral about the fact that pregnant females positive for Hepatitis B should abort their babies as they would give birth to an HBV-positive infant.

**Table 4:** Attitude toward hepatitis B and C status among the study participants (N=756)

Sl. no.	Attitudes	Strongly disagree, n (%)	Disagree, n (%)	Neutral, n (%)	Agree, n (%)	Strongly agree, n (%)
1	When diagnosed with hepatitis B, would you go for further investigation and treatment?	25 (3.3)	30 (3.96)	110 (14.51)	286 (37.73)	307 (40.5)
2	Do you think you can get hepatitis B?	37 (4.88)	89 (11.74)	188 (24.8)	311 (41.03)	133 (17.55)
3	Do you think healthy people need vaccination against hepatitis B?	23 (3.03)	42 (5.54)	157 (20.71)	299 (39.45)	237 (31.27)
4	Do you think hepatitis B vaccination is not effective at your age?	112 (14.78)	233 (30.74)	273 (36.02)	105 (13.85)	35 (4.62)
5	Do you think hepatitis B vaccination is safe?	25 (3.3)	32 (4.22)	239 (31.53)	268 (35.36)	194 (25.59)
6	I feel hepatitis B and C can spread through shaking hands and hugging and kissing the infected person	124 (16.36)	214 (28.23)	293 (38.65)	92 (12.14)	35 (4.62)
7	I feel pregnant women positive for hepatitis B should abort their babies as they will give birth to an HBV-positive infant	97 (12.8)	175 (23.09)	335 (44.2)	102 (13.46)	49 (6.46)

Practices related to hepatitis B and C status

On enquiring about the practices (Table 5), about 24.51% had been screened for hepatitis B or C at least once. Around 67.85% of the individuals reported that they always asked their barber to change the blade before shaving/haircuts. More than half (59.68%) had never checked their antibody titer for hepatitis B.

**Table 5:** Practices related to hepatitis B and C among the study participants (N=759)

Sl. no.	Practices	Never, n (%)	Rarely, n (%)		Sometimes, n (%)	Often, n (%)	
1	How often do you ask your barber to change the blade for safe equipment for shaving/haircuts?	53 (6.98)	52 (6.85)		67 (8.83)	72 (9.49)	
2	Do you avoid meeting/maintaining a distance from hepatitis B patients?	314 (41.37)	100 (13.18)		106 (13.97)	92 (12.12)	
		No, n (%)		Not sure, n (%)			Yes, n (%)	
3	Have you ever been screened for hepatitis B/C?	356 (46.9)		217 (28.59)			186 (24.51)	
4	Have you vaccinated yourself against hepatitis B?	329 (43.35)		174 (22.92)			256 (33.73)	
5	Have you ever checked your antibody titer for hepatitis B?	453 (59.68)		175 (23.06)			131 (17.26)	

Awareness regarding hepatitis B and C status

**Figure 1:**
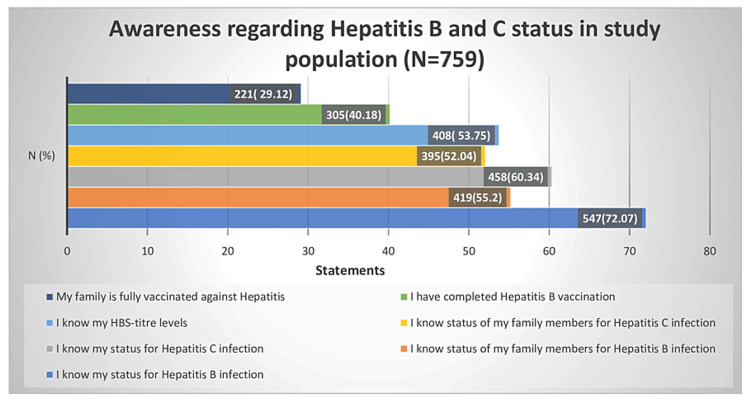
Awareness regarding hepatitis B and C status among the study participants (N=759)

The level of awareness regarding hepatitis B and C status in the study population is illustrated in Figure 1. The values represent the proportion of respondents who affirmed each statement, highlighting gaps in knowledge and vaccination coverage for hepatitis B and C within the study population. The majority (72.07%) of the individuals were aware of their hepatitis B status and more than half (55.2%) were aware of their family’s viral hepatitis status. Around 40.18% of the individuals had completed the hepatitis B vaccination course.

Association between knowledge and demographic factors

As shown in Table 6, based on multivariate analysis, age, gender, and education were significant factors (p<0.001) associated with knowledge (poor/good). Compared to males, females had 2.36 higher AOR (95% CI: 1.66-3.37) regarding knowledge about hepatitis B and C. As the level of education increased (AOR: 2.88, 95% C.I: 1.85-4.48), knowledge of hepatitis B and C also increased. Socioeconomic status was significantly associated (p<0.001) whereas family type was not found to be a factor statistically associated with knowledge regarding hepatitis B and C.

**Table 6:** Association between knowledge and demographic factors *P-value of the model is <0.001. Pseudo R^2^ is 0.2130 AOR: adjusted odds ratio; CI: confidence interval

Demographic characteristics	Poor knowledge, n (%)	Good knowledge, n (%)	OR (95% CI)	P-value (chi-square test)	AOR (95% CI)	*P-value (chi-square test)
Age						
<35 years	240 (48.39)	256 (51.61)	1.92 (1.41–2.61)	<0.001	1.84 (1.27–2.66)	0.001
≥35 years	169 (64.26)	94 (35.74)	1		1	
Gender						
Male	282 (63.37)	163 (36.63)	1	<0.001	1	<0.001
Female	127 (40.45)	187 (59.55)	2.55 (1.89–3.43)		2.36 (1.66–3.37)	
Education						
Intermediate or less	175 (82.55)	37 (17.45)	1	<0.001	1	<0.001
Diploma and above	234 (42.78)	313 (57.22)	6.33 (4.27–9.37)		2.88 (1.85–4.48)	
Job sector						
Dependent	66 (75.86)	21 (24.14)	1	<0.001	1	
Unemployed	58 (57.43)	43 (42.57)	2.33 (1.24–4.37)		1.98 (0.98–3.99)	0.056
Self-employed	28 (62.22)	17 (37.78)	1.91 (0.87–4.15)		2.87 (1.19–6.94)	0.019
Government	68 (29.82)	160 (70.18)	7.39 (4.19–13.04)		5.19 (2.70–9.95)	<0.001
Private	176 (64.71)	96 (35.29)	1.71 (0.98–2.97)		1.87 (0.99–3.54)	0.053
Public	13 (50.00)	13 (50.00)	3.14 (1.26-7.83)		2.58 (0.92–7.23)	0.071
Type of family						
Living alone	28 (62.22)	17 (37.78)	1	0.123		
Nuclear	243 (52.03)	224 (47.97)	1.52 (0.81–2.84)		-	-
Joint	129 (54.66)	107 (45.34)	1.36 (0.71–2.63)			
Extended	9 (81.82)	2 (18.18)	0.37 (0.07–1.90)			
Socioeconomic status (as per BG Prasad classification 2021), ₹						
Class I (>7770)	165 (40.05)	247 (59.95)	3.10 (1.59–6.04)	<0.001	1.94 (0.93–4.04)	0.075
Class II (3808–7770)	70 (54.26)	59 (45.74)	1.75 (0.84–3.61)		1.75 (0.80–3.86)	0.164
Class III (2253–3808)	84 (77.78)	24 (22.22)	0.59 (0.27–1.29)		0.65 (0.28–1.50)	0.311
Class IV (1166–2253)	61 (91.04)	6(8.96)	0.20 (0.07–0.58)		0.33 (0.11–1.00)	0.052
Class V (<1166)	29 (67.44)	14 (32.56)	1		1	

Correlation between knowledge, attitude, and practice

As seen in Table 7, knowledge showed a significant positive correlation with awareness (r=0.25), attitude (r=0.49), and practice (r=0.34).

**Table 7:** Correlation between knowledge, attitude, and practice *Significant p<0.05. Pearson's correlation regarding knowledge, attitude, and practice

	Knowledge	Awareness	Attitude	Practice
Knowledge	1.00			
Awareness	0.2516*	1.00		
Attitude	0.4987*	0.1986*	1.00	
Practice	0.3427*	0.4597*	0.3793*	1.00

## Discussion

Our survey results indicate that the general public in India has a moderate level of knowledge about HBV and HCV, with an average score of 19 ± 5.65 out of a maximum score of 45 with significant gaps in understanding specific transmission routes and preventive measures. The comprehensive survey design helped us to assess knowledge across four domains: general overview of hepatitis (including the organ affected, types of hepatitis, cause of disease and method of spread, signs and symptoms, treatment, and vaccination), mode of transmission, prevention by vaccine, and complications and treatment.

Viral hepatitis poses a significant global public health challenge. Hepatitis B and C, in particular, place a heavy burden on healthcare systems and are leading causes of hepatocellular carcinoma, resulting in substantial morbidity and mortality [12]. Inadequate knowledge about hepatitis B and C among the general population has significant public health implications. Understanding these implications is crucial for developing effective strategies to combat the spread and impact of these infections. Insufficient knowledge about the modes of transmission of hepatitis B and C can lead to higher transmission rates. Individuals unaware of how the virus spreads may not take necessary precautions, such as avoiding sharing needles, practicing safe sex, or ensuring safe blood transfusions. Lack of awareness about the symptoms and severity of hepatitis can result in delayed diagnosis and treatment. Poor knowledge can lead to a higher prevalence of these infections, increasing healthcare costs related to treating liver cirrhosis, liver cancer, and other complications. Moreover, misconceptions about hepatitis can contribute to stigmatization and discrimination against those infected.

Our study results align with a similar study that reported suboptimal knowledge, with only 25% of the respondents demonstrating good knowledge (≥12 points) and 44% having either poor or incorrect knowledge [11]. In our study, the respondents had a mean age of 33.4 ± 11.5 years with a male dominance (58.6%); in another study, 43% of respondents were male, with the majority aged between 20 to 25 years, with a satisfactory level of awareness about HBV but notably lesser for HCV among the participants [13]. In this study, participants were knowledgeable about the causative organisms, body organs affected by the disease, and the risks associated with tattooing and contaminated needles and mother-to-child transmission for hepatitis B and C and adequately understood its complications. However, they lacked knowledge in terms of identifying types of hepatitis, the curability of the infection, and the vaccination schedule for hepatitis B.

In contrast with other studies, participants in our study recognized blood transfusions, unsterilized syringes, and barber blades as major transmission modes for hepatitis B and C. Still, they were uncertain about the risks associated with tattooing and ear/nose piercing [14]. In the current study, knowledge about the hepatitis B vaccine and its dose and schedule was found to be unsatisfactory among the participants. In another study, 40% of the sample knew about the availability of the vaccination, and only 20% had been vaccinated themselves. The most common reason for not getting vaccinated was a lack of awareness [15]. Most participants in the study were aware of their own and their family's hepatitis B and C status, but many had not completed the full vaccination course. Only 29% of their family members were fully vaccinated against the disease. However, in another study, just 11.3% of the females had been tested for HBV or HCV in the year before the survey [16]. Although nationwide vaccination campaigns aim to reach this underrepresented population, screening family members of Asian patients with HBV remains a significant challenge [17].

The mean attitude-related score among our participants was 15.28 ± 4.13. A significant majority (78.73%) expressed a willingness to seek further investigation and treatment if diagnosed with hepatitis B, though only 17.55% strongly believed they were at risk of contracting the disease. Comparatively, in a national survey, the mean attitude score was 4.88 ± 4.38 (range: -20 to +20) [18]. The present study results also contrast with some other studies where 57% of participants reported poor practice [19]. The difference may be attributed to the varying socioeconomic characteristics of the populations included in the two studies. However, the confidence intervals for most estimates from previous studies overlap with those of the present study.

Our findings indicate that age, gender, and education are significant factors (p<0.001) associated with good knowledge. Compared to males, females were reported to have 2.36 higher odds of knowledge about hepatitis B and C. As the level of education increased, knowledge of hepatitis B and C also increased, aligning with previous research [20]. Type of family was not found to be a factor associated with knowledge regarding hepatitis B and C, as in a similar study [21]. The study revealed moderate knowledge about hepatitis among the general population, with significant gaps in understanding about specific transmission routes and preventive measures. Attitudes toward vaccination and treatment were generally positive, albeit with notable misconceptions, such as the belief that healthy individuals do not need vaccination. Practices related to hepatitis prevention, such as vaccination and screening, were suboptimal, revealing a more in-depth understanding of respondents' knowledge gaps and areas of strength.

The present study findings highlight the need for conducting longitudinal studies to examine changes in knowledge, attitude, and practice over time, adopting qualitative research methods to explore the reasons behind misconceptions and poor practices, and performing comparative studies involving different demographic groups to identify specific needs and tailor interventions accordingly. Given the ongoing pandemic, leveraging vaccination centers to disseminate health information could be a strategic approach to improving hepatitis awareness and prevention.

Recommendations

To address the gaps in knowledge and vaccination coverage for hepatitis B and C, several recommendations are proposed. Firstly, implementing targeted educational campaigns is essential to enhance understanding of hepatitis transmission and prevention. Second, increasing efforts to promote hepatitis B vaccination, especially among high-risk groups, is crucial. Third, expanding accessibility and raising awareness of hepatitis B and C screening services can significantly improve early detection and management. Lastly, ensuring healthcare providers have the necessary tools and information will enable them to educate patients more effectively. By focusing on these areas, public health initiatives can better equip individuals with the knowledge and resources needed to prevent and manage hepatitis B and C.

Limitations

The limitations of this study include the reliance on self-reported data, which may have introduced response bias and thereby affected the accuracy of the results. Additionally, the study was conducted at a single COVID-19 vaccination center in a tertiary care hospital, which may limit the generalizability of the findings to the broader population.

## Conclusions

Our survey results indicate that the general public in India has a moderate level of knowledge about HBV and HCV. This study highlights the need for targeted educational campaigns to improve knowledge about hepatitis transmission, symptoms, and prevention. To reach a broad audience, these campaigns should use various media channels, including social media, television, and print. The engagement of community leaders and organizations in spreading awareness and promoting positive attitudes toward hepatitis prevention and treatment needs to be leveraged. Community-based participatory approaches can enhance the reach and effectiveness of public health interventions.

The findings also underscore the need for targeted educational interventions to address knowledge gaps and correct misconceptions about hepatitis B and C. Public health education should be leveraged to not only raise awareness but also to empower individuals with the knowledge needed to adopt preventive practices. This, in turn, can help to reduce the overall burden of hepatitis in the community. The study emphasizes that a multifaceted approach, combining media outreach with community engagement and participatory methods, is essential for the successful dissemination of information and the promotion of healthy behaviors.
